# Supplementary material for: Emergent representations of graphical structure in mechanistic neural models of causal judgment
Source: bioRxiv. 2026 May 15:2026.05.13.724819. Preprint. [Version 1] doi: 10.64898/2026.05.13.724819 (PMC13192587; doi:10.64898/2026.05.13.724819)
Supplement: Supplement 1 [file NIHPP2026.05.13.724819v1-supplement-1.pdf]

bioRxiv preprint doi: https://doi.org/10.64898/2026.05.13.724819; this version posted May 15, 2026. The copyright holder for this preprint (which was not certified by peer review) is the author/funder, who has granted bioRxiv a license to display the preprint in perpetuity. It is made available under a CC-BY 4.0 International license.

Supplementary figures

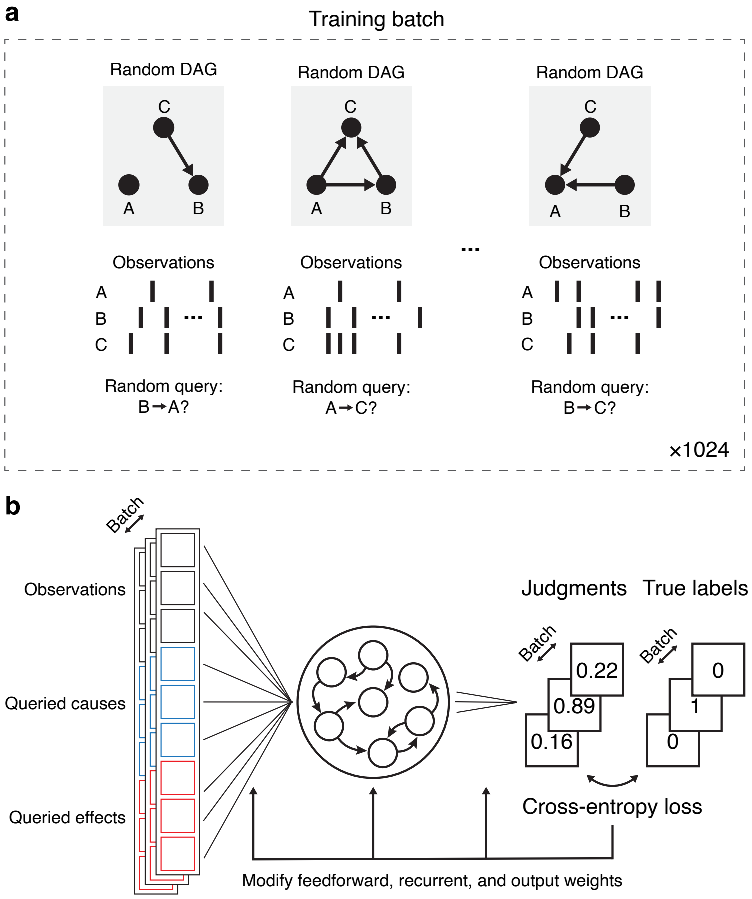

Figure S1: Overview of RNN training paradigm. **a**, A batch consists of 1024 training examples. Each example is made up of a sequence of observations from a randomly sampled DAG, a random query, and the true label (0 or 1 corresponding to whether the query is true or false). **b**, The RNN generates numerical judgments about whether the query is true or false, for all examples in the batch simultaneously, which are compared to the true labels and used to adjust the feedforward, recurrent, and output weights via error backpropagation.

bioRxiv preprint doi: https://doi.org/10.64898/2026.05.13.724819; this version posted May 15, 2026. The copyright holder for this preprint (which was not certified by peer review) is the author/funder, who has granted bioRxiv a license to display the preprint in perpetuity. It is made available under a CC-BY 4.0 International license.

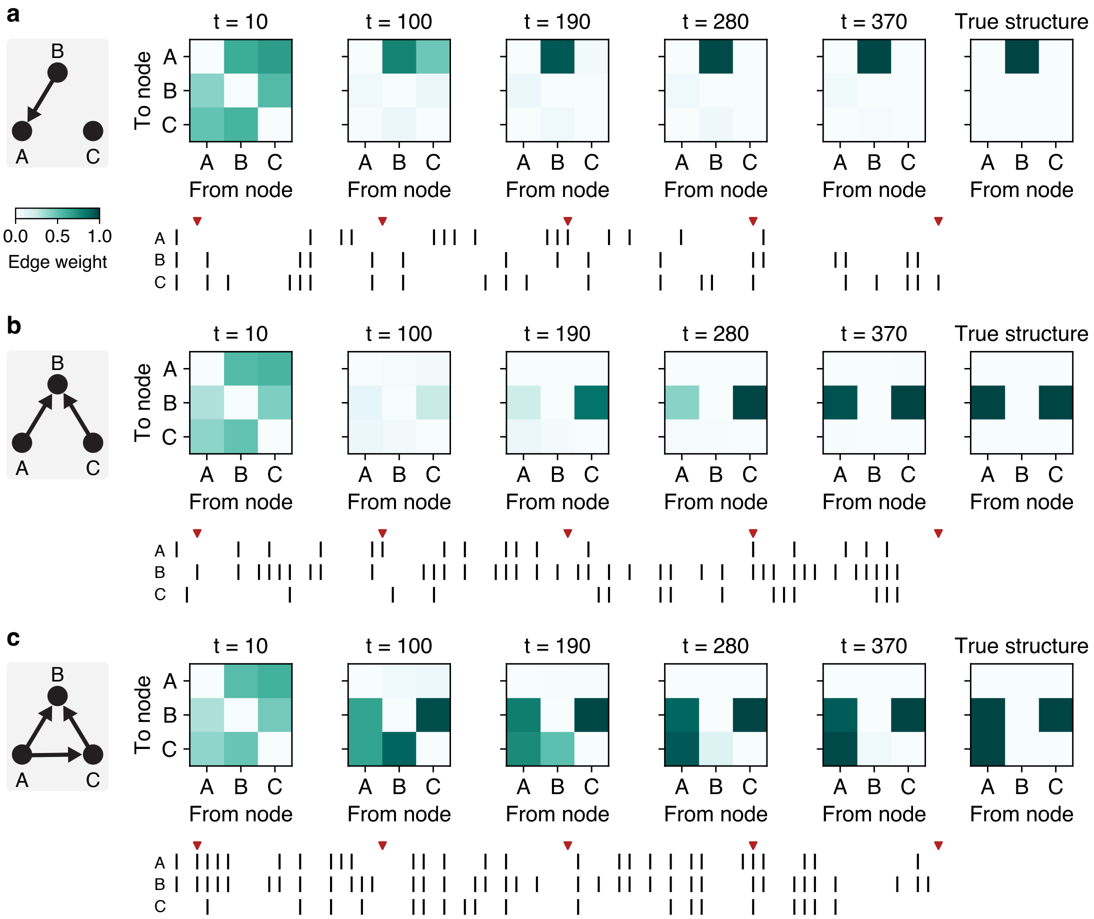

Figure S2: Additional examples of how RNNs' beliefs about complete DAG structures evolve through time, for a singleton DAG (**a**), a collider DAG (**b**), and a mediation DAG (**c**).

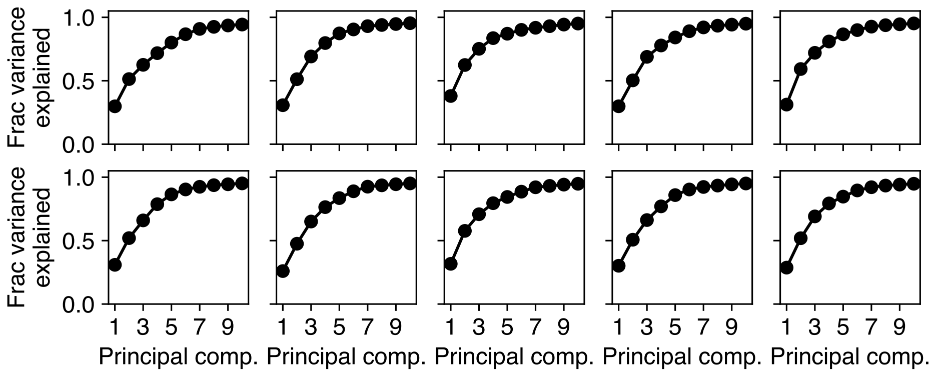

Figure S3: Cumulative variance explained by the first 10 principal components, across 10 randomly initialized RNNs trained on the 3-node DAG task.

bioRxiv preprint doi: https://doi.org/10.64898/2026.05.13.724819; this version posted May 15, 2026. The copyright holder for this preprint (which was not certified by peer review) is the author/funder, who has granted bioRxiv a license to display the preprint in perpetuity. It is made available under a CC-BY 4.0 International license.

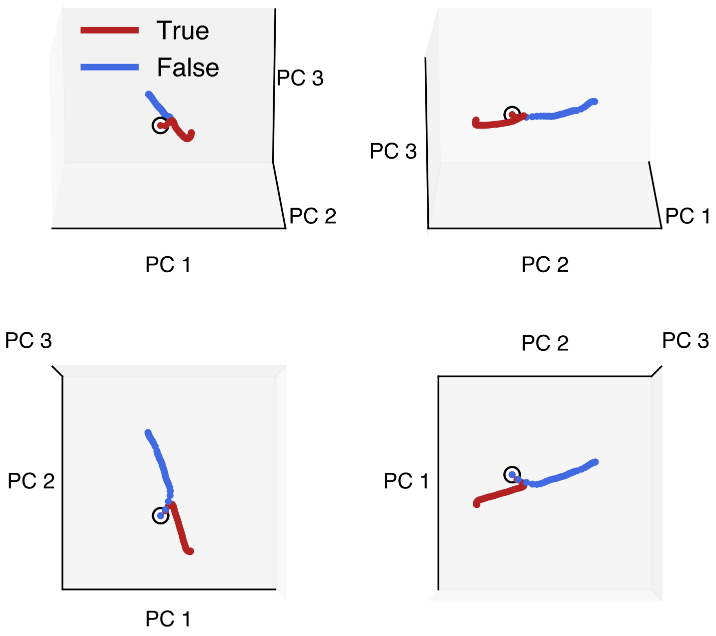

Figure S4: Decision axis (i.e. “judgment axis"), derived from neural activity by averaging trajectories corresponding to DAG edges that exist (“True", red) vs edges that do not exist (“False", blue). Averages are taken over 50 samples from each of the 25 possible DAGs on three variables. Cf. Figure 6 for a direct visualization of the decision axis in the top principal components via projection of the RNN readout weights.

bioRxiv preprint doi: https://doi.org/10.64898/2026.05.13.724819; this version posted May 15, 2026. The copyright holder for this preprint (which was not certified by peer review) is the author/funder, who has granted bioRxiv a license to display the preprint in perpetuity. It is made available under a CC-BY 4.0 International license.

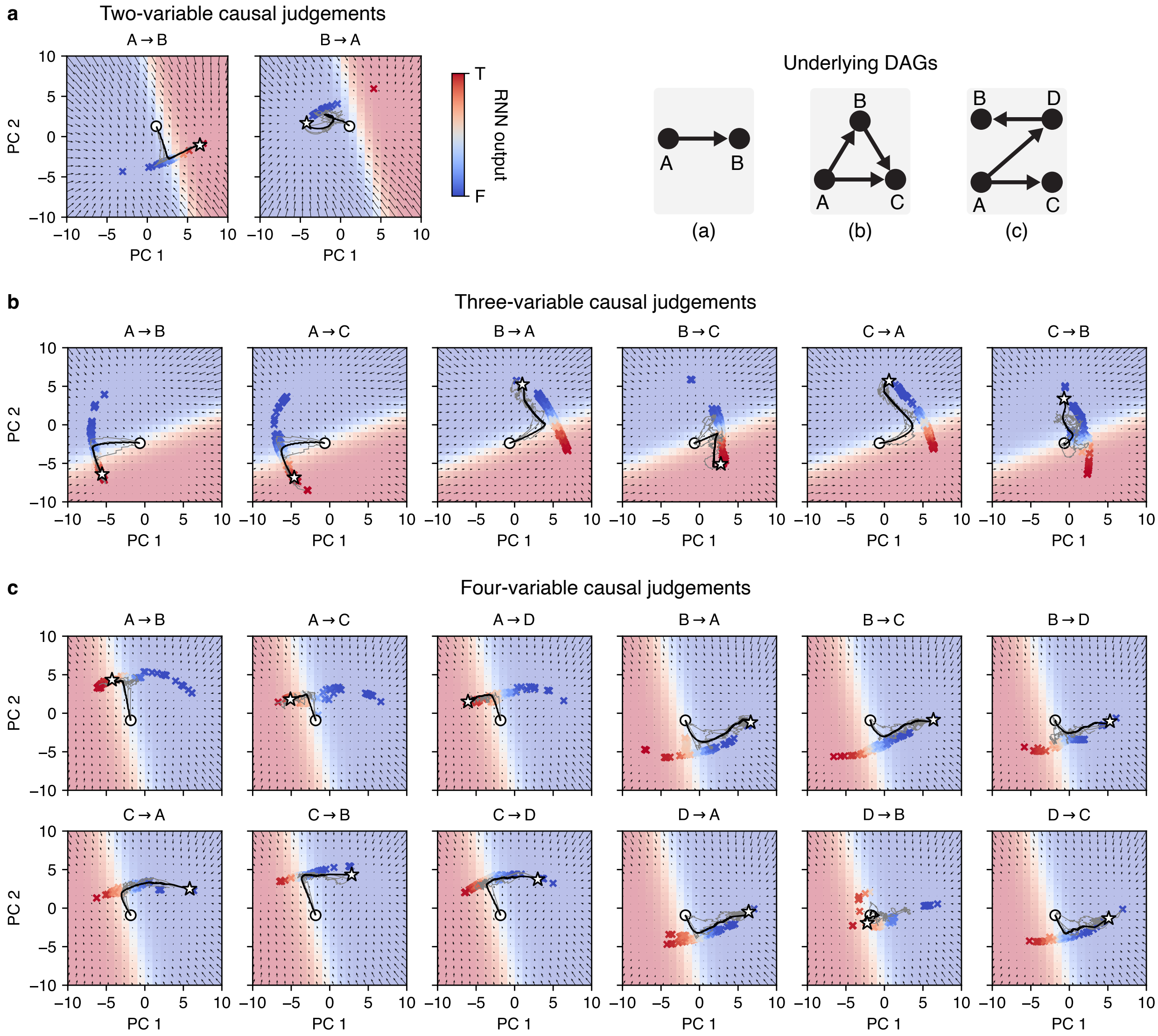

Figure S5: Comparison of fixed point structure from DAGs on two (**a**), three (**b**), and four (**c**) nodes shows line attractor dynamics represent a general mechanism for performing causal judgments.

bioRxiv preprint doi: https://doi.org/10.64898/2026.05.13.724819; this version posted May 15, 2026. The copyright holder for this preprint (which was not certified by peer review) is the author/funder, who has granted bioRxiv a license to display the preprint in perpetuity. It is made available under a CC-BY 4.0 International license.

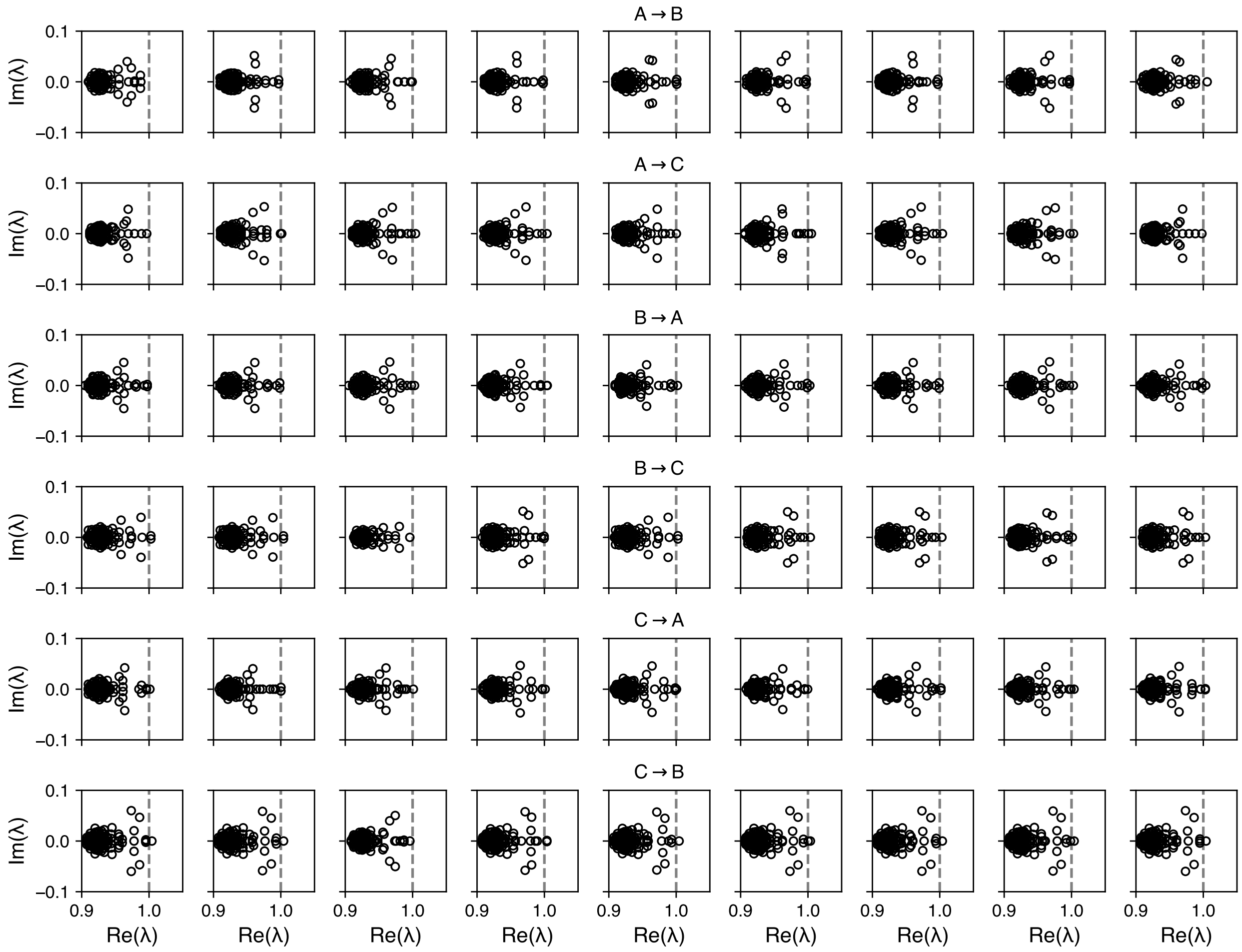

Figure S6: Eigenvalue spectra corresponding to nine randomly chosen fixed points from each of the six line attractors from Figure 5.

bioRxiv preprint doi: https://doi.org/10.64898/2026.05.13.724819; this version posted May 15, 2026. The copyright holder for this preprint (which was not certified by peer review) is the author/funder, who has granted bioRxiv a license to display the preprint in perpetuity. It is made available under a CC-BY 4.0 International license.

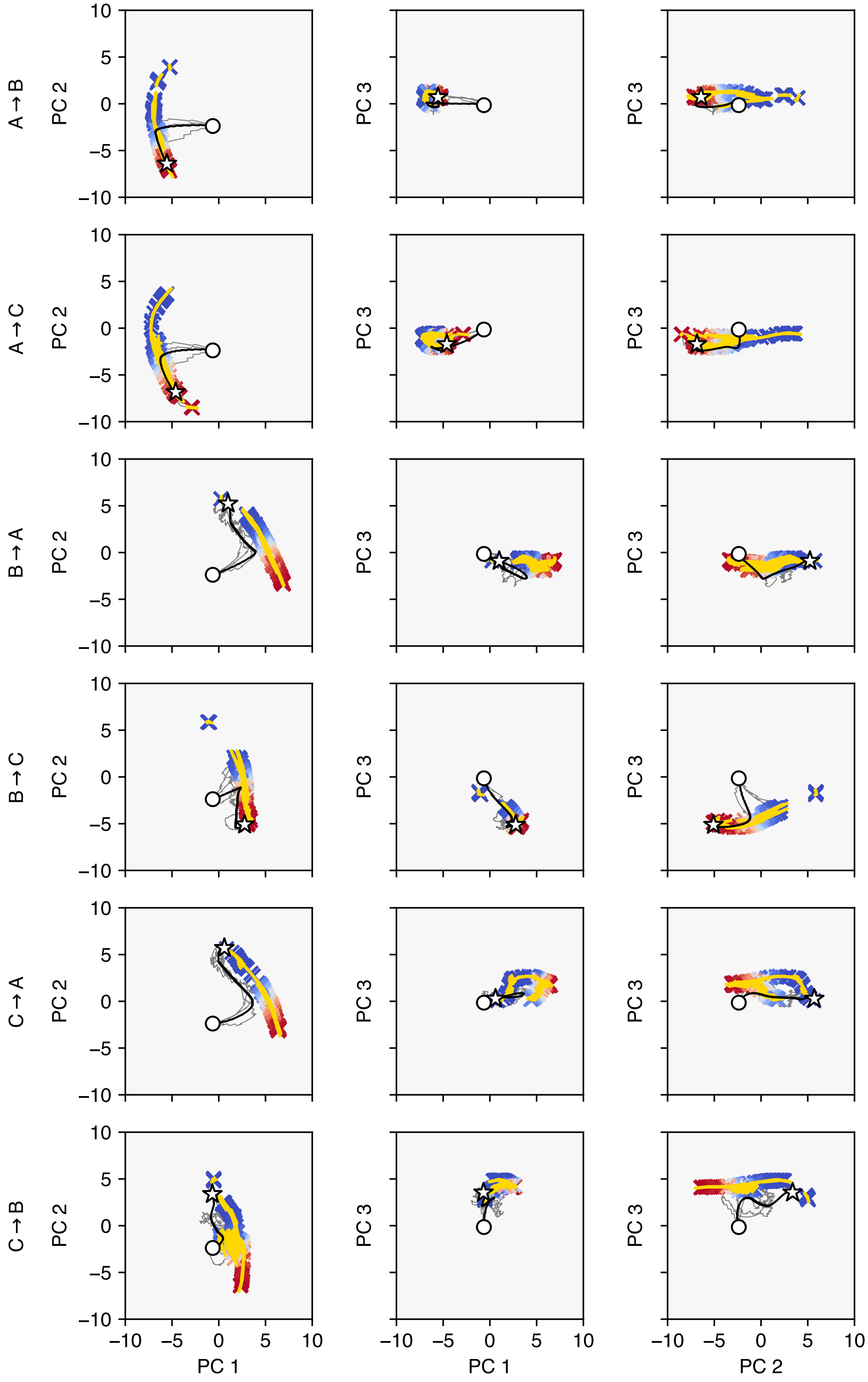

Figure S7: Eigenvectors (yellow lines) associated with the largest real eigenvalues align with the principal direction of the line attractor (red/blue crosses). White circles show trajectory initial points, white stars show trajectory end points. Black lines show averages over 500 trajectories. Faint gray lines show three example individual trajectories.

bioRxiv preprint doi: https://doi.org/10.64898/2026.05.13.724819; this version posted May 15, 2026. The copyright holder for this preprint (which was not certified by peer review) is the author/funder, who has granted bioRxiv a license to display the preprint in perpetuity. It is made available under a CC-BY 4.0 International license.

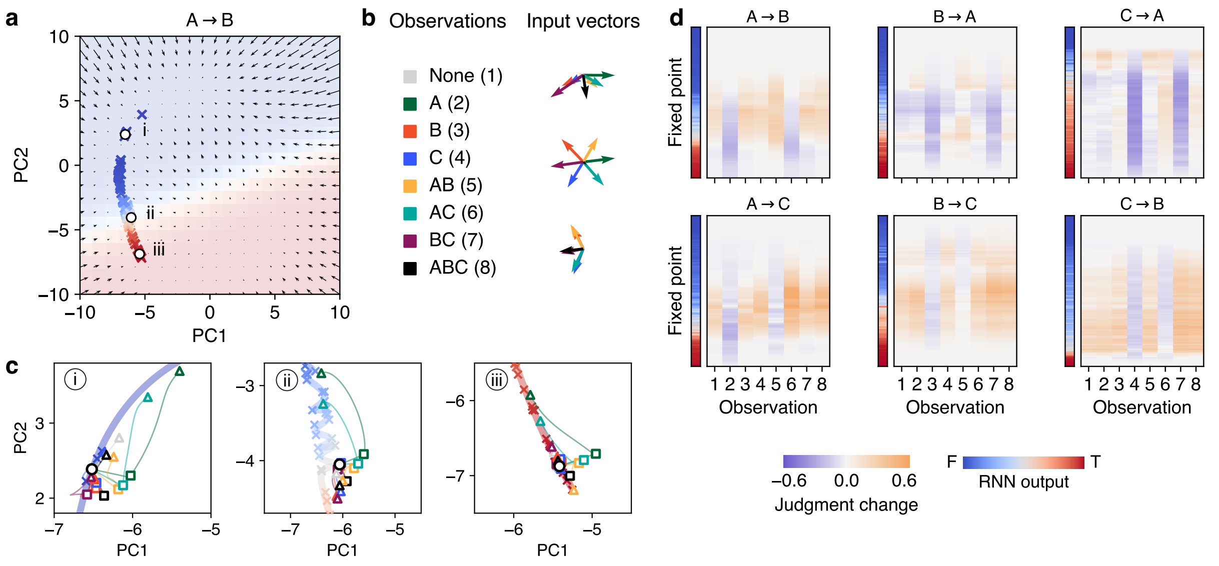

Figure S8: Characterization of how different inputs elicit judgment changes. **a**, Three example fixed points along the $A \rightarrow B$ line attractor. **b**, Left: Colors representing each observation. Right: Input vectors associated with each observation. **c**, Additional examples of the interaction between input vectors and recurrent dynamics, corresponding to the fixed points in (a). **d**, Change in judgment resulting from each observation when the neural state is initialized at a given fixed point. Sufficient time is given for the recurrent dynamics to carry the neural state back to the line attractor. A diversity of state-dependent input effects are revealed through this analysis, that together describes the way in which an RNN makes its judgments. For example, when resolving whether A causes B in the case of the fork DAG with edges $\{(C, A), (C, B)\}$, observing AC almost always moves neural state towards the "false" end of the line attractor. Observing BC has a more subtle effect, however, as the observation pushes the neural state towards "true" if the RNN is uncertain, but pushes the neural state back towards "false" if it already believes that A does cause B. The RNN therefore must rely on instances of AB to confirm that A causes B, whereas instances of A and AC (the candidate cause without the effect) strongly push the RNN towards "false."
